# Response Surface Methodology for the Optimization of Flavan-3-ols Extraction from Avocado By-Products via Sonotrode Ultrasound-Assisted Extraction

**DOI:** 10.3390/antiox12071409

**Published:** 2023-07-11

**Authors:** María del Carmen Razola-Díaz, Vito Verardo, Eduardo Jesús Guerra-Hernández, Belén García-Villanova Ruiz, Ana María Gómez-Caravaca

**Affiliations:** 1 Department of Nutrition and Food Science, University of Granada, Campus of Cartuja, 18011 Granada, Spain; carmenrazola@ugr.es (M.d.C.R.-D.); ejguerra@ugr.es (E.J.G.-H.); belenv@ugr.es (B.G.-V.R.); 2 Institute of Nutrition and Food Technology ‘José Mataix’, Biomedical Research Center, University of Granada, Avda. del Conocimiento s/n, 18100 Granada, Spain; anagomez@ugr.es; 3 Department of Analytical Chemistry, Faculty of Sciences, University of Granada, Avda. Fuentenueva s/n, 18071 Granada, Spain

**Keywords:** avocado seed, avocado peel, flavan-3-ols, sonotrode, Box–Behnken, procyanidins

## Abstract

Avocado peel and seed are the main by-products of avocado processing and are considered as promising sources of phenolic compounds with biological activities. Thus, this research focuses on the establishment, for the first time, of ultrasound-assisted extraction of flavan-3-ols with high antioxidant activity from avocado peel and seed using a sonotrode. Indeed, 2 Box–Behnken designs were performed for 15 experiments, with each design having three independent factors (ratio ethanol/water (*v*/*v*), time (min) and amplitude (%)). In both models, the responses included total procyanidins (flavan-3-ols) measured via HPLC-FLD and antioxidant activity measured via DPPH, ABTS and FRAP. The results showed that applying the sonotrode extraction method could increase flavan-3-ols recovery by 54% and antioxidant activity by 62–76% compared to ultrasound bath technology. Therefore, this technology was demonstrated to be a non-thermal, low time-consuming and scalable method that allowed the recovery of flavan-3-ols from avocado by-products that could be used as functional ingredients.

## 1. Introduction

Avocado is a tropical and subtropical fruit classified in the family *Lauraceae*, genus *Persea*, and species *P. americana*. There are numerous cultivars of avocado around the world, whose specific traits vary depending on the climate in which they are grown, that have different shapes, flavours, textures, colours and smells. The most well-known and marketed types are the ‘Hass’ and ‘Fuerte’ cultivars. Avocado is known for its high nutritional content and health benefits, which are largely due to the fact that avocados are a source of fat-soluble nutrients or phytochemicals [1]. Nowadays, the consumption of avocado is increasing due not only to its nutritional and organoleptic properties, but also to the increased availability of avocado-based products, such as guacamole, oil, jams, juices, ice creams, sauces, etc [2]. In addition, in 2020, total avocado production of 8.06 million tons was reported worldwide, which was 35% higher than in 2015; avocado was the main produced in Mexico, the Dominican Republic, Peru and Colombia [3]. For avocado fruit, only up to 65–73% of its weight is consumed (pulp), and the remaining fruit is discarded as wastes. These wastes are mainly composed of peel and seed, which account for 21–39% of the whole avocado [2], i.e., a by-product generation from 1.24 to 2.5 million tons in 2020. In this context, the valorisation of those by-products is an important environmental problem that must be faced. Until now, avocado by-products have been processed using different biorefinery approaches, such as obtaining bioenergy, biogas, biofuel, biodiesel or bioethanol, as well as the extraction of oils, fermentable sugars, starches, proteins, colorants or phenolic compounds [2]. Avocado peel and seed are considered as promising sources of phenolic compounds and have been reported to possess anti-carcinogenic, anti-inflammatory, anti-microbial and antioxidant properties, among others [4,5,6,7,8]. Specifically, flavan-3-ols from avocado by-products have demonstrated that they possess anticancer activity against melanoma cells [9], can inhibit *Helicobacter pylori* [10] and can improve colonic homeostasis in protein rich diets [11]. Moreover, avocado procyanidins have been reported to inhibit the enzyme polyphenol oxidase, which plays a critical role in phenolic degradation, browning, development of insects, and melanogenesis in food [12]. This result indicates that procyanidins from avocado by-products could be of huge relevance in the fields of nutraceuticals and food. Some authors have reported the extraction of total phenolic compounds from avocado peel and seed via traditional methods, such as maceration, and via innovative methods, such as microwaves, ultrasound bath- or pressurized fluid-assisted extractions; however, there has been no focus on any compound. Thus, the aim of this work is to establish the best method of ultrasound-assisted extraction via sonotrode, which is a technology that could be industrially scaled up to obtain enriched extracts of procyanidins (flavan-3-ols) from avocado peel and seed, which also present high antioxidant activity.

## 2. Materials and Methods

### 2.1. Chemicals and Reagents

The reagents used for the antioxidant assays and catechin standard were purchased from Sigma-Aldrich (St. Louis, MO, USA). Double-deionized water was acquired from Millipore (Bedford, MA, USA). HPLC-grade water, acetic acid, acetonitrile and methanol were purchased from Merck KGaA (Darmstadt, Germany).

### 2.2. Samples

Avocado by-products from the variety ’Hass’ were obtained from a local company located near to the subtropical coastal area of the province of Málaga (Vélez-Málaga, Spain) in October 2020. We separately acquired the generated by-products from the production of guacamole, peels (moisture ~75%) and seeds (moisture ~45%). The samples were dried at 50 °C, grinded and stored at −18 °C until analysis.

### 2.3. Experimental Design

The obtention of avocado peel and seed extracts enriched in procyanidins with a high antioxidant activity was optimized using Box–Behnken designs. Each design consisted of 15 experiments with 3 levels (−1, 0, 1), which were carried out in duplicate. In both peel and seed models, the independent factors were the ratio of ethanol/water (30/70, 65/45, 100/0% *v*/*v*), time (5, 25, 45 min) and amplitude (20, 60, 100%). The dependent variables were total procyanidins and FRAP, DPPH and ABTS. The variables were adjusted to fit a second-order polynomial equation (Equation (1)), in which the response variable was represented by ϒ, the independent factors were represented by X_i_ and X_j_, and the regression coefficients of the model (interception, linear, quadratic and interaction terms) were β_0_, β_i_, β_ii_ and β_ij_, respectively.

Equation (1) was the second-order polynomial equation.

(1) $$\Upsilon =\beta_{0}+\sum_{i=0}^{4}\beta_{i}X_{i}+\sum_{i=0}^{4}\beta_{ii}X_{ii}^{2}+\sum_{i=0}^{4}\sum_{j=0}^{4}\beta_{ii}X_{i}X_{j}$$

ANOVA analyses were performed in order to evaluate the adjustment of the models. Optimal conditions were established via response surface methodology (RSM).

### 2.4. Sonotrode Extraction

For each trial, 0.5 g of the sample was weighted, and 100 mL of ethanol/water (*v*/*v*) were added in 250 mL beakers. After that, the sonotrode (UP400St ultrasonic processor, Hielscher, Teltow, Germany), for which the amplitude was previously configured, was introduced into the beaker and the sample was sonicated during the established time. The pulse was locked at 100% for all analyses. Once the analysis time finished, the content of the beaker was verted to a Falcon tube, and it was centrifuged for 10 min at 8603× *g* and 0 °C. The supernatants were collected, evaporated, and reconstituted in 2–4 mL of methanol/water (1:1, *v*/*v*). The final extracts were filtered using cellulose acetate filters of 0.2 mm (Millipore, Bedford, MA, USA) and stored at −18 °C until later analysis.

### 2.5. Ultrasonic Bath Extraction

The ultrasonic bath extraction was carried out following the protocol previously used by López-Cobo et al. [13], albeit with slight modifications. In brief, 2 g of sample powder was extracted with 15 mL of ethanol/water (80:20, *v*/*v*) in 50 mL Falcon tubes. The mixture was placed in an ultrasonic bath (Sonorex RK 52, Bandelin, Berlin, Germany) for 15 min and then centrifuged for 10 min at 8603× *g* and 0 °C. The supernatant was removed, and the extraction was twice repeated. The supernatants were collected, evaporated, and reconstituted in 3 mL of methanol/water (50:50, *v*/*v*). The final extracts were filtered using cellulose filter of 0.2 mm (Millipore, Bedford, MA, USA) and stored at −18 °C until later analysis.

### 2.6. Determination of Procyanidins via HPLC-FLD-MS

The methodology used in the determination of flavan-3-ols was previously reported by López-Cobo et al. [13]. For the analysis, an Agilent 1200 Series (Agilent Technologies, Palo Alto, CA, USA), which was equipped with a quaternary pump delivery system, a degasser, an autosampler and a fluorometric detector (FLD), was used. It used a Develosil Diol 100 Å column that had the following dimensions: 5 µm and 250 × 4.6 mm ID (Phenomenex, Torrance, CA, USA). Mobile phases A and B consisted of acidic acetonitrile ((A) CH_3_CN:CH_3_COOH, 98:2; *v*/*v*) and acidic aqueous methanol ((B) and CH_3_OH:H_2_O:CH_3_COOH, 95:3:2; *v*/*v*/*v*), respectively. The starting mobile phase condition was 0% B, which was held for 10 min. Next, the gradient elution was 0% B for 5 min; B was increased to 38% over 50 min, and then to 100% over 3 min. Next, 100% B was maintained for 10 min and then returned to 0% B over 6 min and maintained for 10 min. An excitation wavelength of 230 nm and an emission wavelength of 321 nm were fixed for the fluorescence detection. The injection volume was 10 µL. The identification of flavan-3-ols was performed according to data previously described in the literature [13], with elution occurring according to the degree of polymerization, with the monomers eluting first, followed by the different oligomers [14]. A standard curve of catechin at 6 concentration levels ranging from 10 to 650 ppm was carried out for the quantification of flavan-3-ols (y = 3137.9x − 9865.6, R^2^ = 0.999982). In addition, correction factors suggested by Robbins et al. [14] were used for quantification. Results are expressed as mg catechin equivalents (CE)/g d.w.

### 2.7. Antioxidant Assays: ABTS, DPPH and FRAP Methods

The antioxidant capacity was evaluated twice in all experiments and via three different methods: ABTS, DPPH and FRAP. The ABTS method was carried out by adding 1 mL of the ABTS solution into 100 µL of extract, and the detriment of absorbance during 30 min at 734 nm was measured [15]. For DPPH, 2.9 mL of DPPH were added to 100 µL of each extract, and the absorbance was measured for 30 min at 517 nm [16,17]. FRAP assay was performed by adding 90 µL of distilled water and 900 µL of the FRAP reagent to 30 µL of each extract, which were kept in darkness for 30 min at 37 °C and measured at 595 nm [18]. For all assays, the measurements were performed via a UV–visible spectrophotometer (Spectrophotometer 300 Array, UV–Vis, single beam, Shimadzu, Duisburg, Germany). They used standard curves of Trolox (1, 5, 10, 20, 50, 80, 100, 150, 200 ppm). Results are expressed as mg Trolox equivalents (TE)/g d.w.

### 2.8. Data Elaboration

The software used for the simulations and statistical analyses, such as ANOVA and statistical differences (Tukey test), was Statistica 8.0 package (StatSoft, Tulsa, OK, USA). Metaboanalyst 5.0 software (Xia Lab, McGill, Montréal, QC, Canada) was used to create the Pearson correlation heatmap.

## 3. Results and Discussion

### 3.1. Determination of Procyanidins and Antioxidant Capacity in Avocado By-Products

To optimize the extraction conditions via sonotrode of avocado by-products, two Box–Behnken designs were created, using the total procyanidin content measured via HPLC-FLD and the antioxidant activities measured via three methods (DPPH, ABTS and FRAP) as response variables (Table 1).

Regarding the avocado peel model, the results obtained for procyanidins ranged from 1.05 to 20.02 mg CE/g d.w., while for the antioxidant assays, values ranged from 0.31 to 12.29 µg TE/g d.w. for DPPH, 1.17–23.12 mg TE/g d.w. for ABTS, and 1.05–14.79 mg TE/g d.w. for FRAP technique. In all cases, the lowest values corresponded to the extracts obtained using 100% ethanol as solvent, increasing the content as the time and amplitude of extraction increased. In contrast, the highest value for all variables was obtained using 65% of ethanol.

The total content of procyanidins obtained in the avocado seed model was between 4.29 and 16.09 mg CE/g d.w., and the ranges observed for DPPH, ABTS and FRAP were 1.92–17.11, 1.93–25.77 and 2.36–24.82 mg TE/g d.w., respectively. In the antioxidant assays, the lowest values were obtained for the same extraction conditions: 100% ethanol, 25 min and 20% amplitude. Similar to the avocado peel model, the highest value for all variables was obtained using ethanol/water in the intermedium value tested and at amplitudes between 60 and 100%.

### 3.2. Fitting the Model

The experimental results of the avocado peel and seed models were adjusted to fit second-order polynomial equations (Equation (1)), and all regression coefficients with the effects and *p* values obtained are shown in Table 2. In all cases, we selected a significance level of *p* < 0.05, the non-significant terms were discarded, and the models were recalculated by considering only the significant terms.

In the avocado peel model, the lineal terms β_1_ and β_2_, the crossed interaction β_23_ and all quadratic terms (β_11_, β_22_, and β_33_) showed significant effects for the four response variables. In addition, the linear term β_3_ and the interaction between ethanol/water and amplitude (β_13_) for procyanidins and DPPH, as well as the interaction between ethanol/water and time β_12_ for ABTS, were significant. According to the effects reported in Table 2, the factor ethanol/water (β_1_) had the highest negative effect in the model, followed by the interaction between time and amplitude (β_23_). In contrast, all quadratic interactions showed positive effects. Moreover, we revealed a high correlation between the response variables and the factors (R^2^ = 0.9996, 0.9997, 0.9635 and 0.9971 for procyanidins, DPPH, ABTS and FRAP, respectively). The ANOVA test revealed the validity of the model, as all *p*-model values were significant, and the lack of fit was non-significant (*p* > 0.05).

Concerning the avocado seed model, almost all linear, interacting, and quadratic terms were found to have significant effect. Only the amplitude (β_3_) for ABTS and the crossed interaction between time and amplitude (β_23_) for FRAP did not present significance. The correlation between factors and all responses was demonstrated because their determination coefficients were higher than 0.9 in all cases (0.9907, 0.9885, 0.9878 and 0.9933 for procyanidins, DPPH, ABTS and FRAP, respectively). The validity of the model was confirmed based on ANOVA finding a lack of fit for *p* values > 0.05 and significative *p*-model values.

### 3.3. Optimization of Sonotrode Extraction Conditions

The response surfaces graphs obtained for each variable were studied to select the optimal conditions for each by-product by establishing a compromise between them, aiming extracts with the highest total procyanidin contents and high antioxidant activity. Moreover, one of the objectives of this study was to develop an optimal sonotrode extraction process that could be industrially scaled up; thus, other reasons, such as economic and energy costs, were considered.

**Figure 1:**
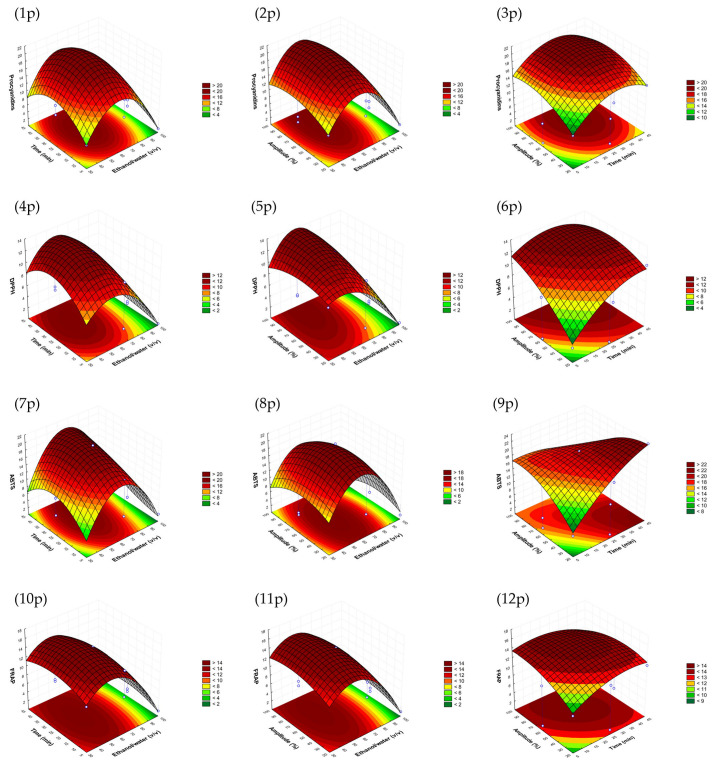
Response surface graphs (1p–12p) of the avocado peel sonotrode model that show the combined effects of the process variables: ethanol/water (*v*/*v*), time (min) and amplitude (%) for procyanidins (mg CE/g d.w.) and DPPH, as well as ABTS and FRAP antioxidant assays (mg TE/g d.w.).

In Figure 1 (from 1p to 12p), it is shown that the highest responses for the avocado peel model could be obtained at an ethanol/water ratio lower than 70:30, *v*/*v* but higher than 40%, with the highest response being near to the intermedium value evaluated (65%). Regarding amplitude, the highest responses were reached between 70 and 100%; thus, amplitude was selected to be as small as possible to reduce energy consumption. The time was also selected to be as short as possible, taking into account the other parameters, to achieve the fastest procedure.

**Figure 2:**
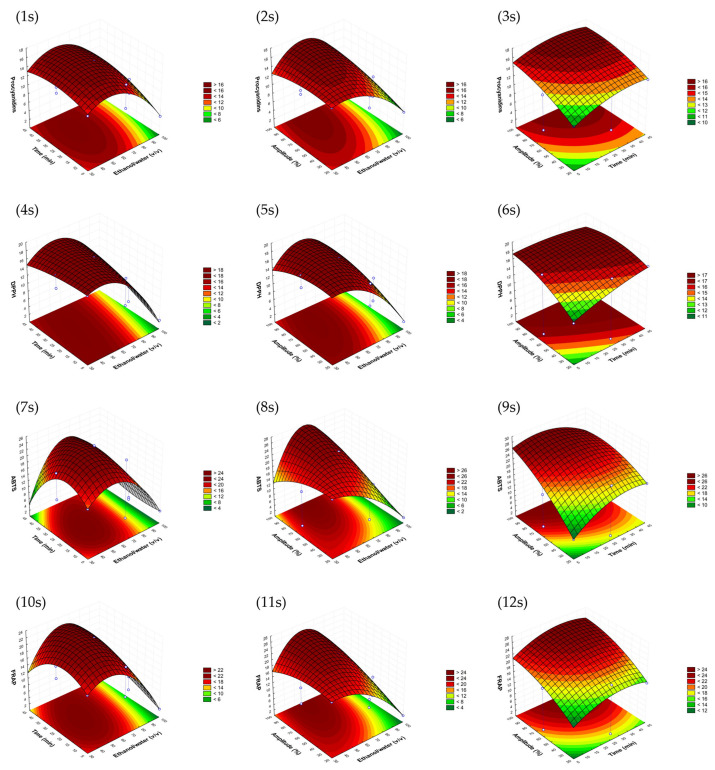
Response surface graphs (1s–12s) of the avocado seed sonotrode model that show the combined effects of the process variables: ethanol/water (*v*/*v*), time (min) and amplitude (%) for procyanidins (mg CE/g d.w.) and DPPH, as well as ABTS and FRAP antioxidant assays (mg TE/g d.w.).

Similar response surfaces were obtained for the avocado seed model, as can be seen in Figure 2. In this case, the ethanol/water ratio selected could be slightly lower than that of the peel. In fact, in terms of economic costs, it is advisable to choose lower percentages of ethanol and higher percentages of water. However, higher amplitudes of 90% seemed to be necessary to obtain better performances. This fact could essentially be attributed to the differences between matrices’ structures. Once again, the time was selected to be as low as possible.

In brief, the optimal conditions selected for the avocado peel model were 60:30, (*v*/*v*) ethanol/water, 30 min and amplitude 70%, while the optimal conditions for the avocado seed model 55:45 (*v*/*v*) ethanol/water, 30 min and amplitude 90% (Table 3).

The accuracy of the two mathematical models was verified (Table 3). In all cases, there were no significant differences between the model’s predicted values and the experimental results obtained based on the optimal conditions. Moreover, the coefficients of variation were lower than five for the total procyanidins and antioxidant assays in peel and seed models.

### 3.4. Comparison between Sonotrode and Ultrasonic Bath-Assisted Extractions

Figure S1 shows a fluorescence chromatogram of the identified flavan-3-ols in avocado peel and seed extracts obtained via sonotrode at optimal conditions. The quantification via HPLC-FLD of procyanidins in both seed and peel extracts obtained via sonotrode and ultrasound bath technology are presented in Table 4.

The total procyanidin content in the avocado peel and seed optimal extracts were 20.8 and 16.69 mg/g d.w., respectively. Total phenolic compounds from avocado peels and seeds measured via spectrophotometry (Folin Ciocalteu) or HPLC-MS have been reported by several authors using hydroethanolic liquid–liquid extraction [19], ultrasound bath [4,20,21], microwave [22,23,24], vacuum microwave [25] and pressurized fluid [26]-assisted extractions. However, no previous references have been found to the use of sonotrode as an ultrasound-assisted extraction technology or the optimization of extraction of procyanidins in avocado peels and seeds. We checked other works related to flava-3-ols analysed via HPLC-FLD in other matrices: in cocoa powders, the total procyanidin content ranged from 3.31 to 28.58 mg/g d.w. [27], from 10.5 to 15.8 mg/g d.w. in *Psidium guayaba* leaves [28], from 0.73 to 22.29 mg/g d.w. in cranberry extracts [29], from 0.29 to 0.65 mg/g d.w. in barley flour [30] and from 3.91 to 14.18 mg/g d.w. in grape seeds [31]. Thus, the obtained results were in concordance. Moreover, the optimal extracts were compared to controls via ultrasonic bath extraction. According to the results shown in Table 4, sonotrode allowed us to obtain a significative (*p* < 0.05) increment, which was 54% higher, in the content of total procyanidins than ultrasound bath in avocado by-products. Furthermore, the avocado peel sonotrode extract had 62, 66 and 67% higher antioxidant activity via DPPH, ABTS and FRAP, respectively, than in the control. Similarly, regarding the avocado seed sonotrode extract, the results were 70, 76 and 70% higher than in the control for DPPH, ABTS and FRAP, respectively. All increments in the antioxidant activity of the extracts obtained via sonotrode were statistically significant (*p* < 0.05) (Figure S2) compared to those obtained via ultrasound bath.

After comparing the antioxidant activities of the optimal extracts with the results obtained by other authors, few references were found. Overall, the results are in the same range of magnitude as the antioxidant activity reported by Figueroa et al. [22], Del Castillo-Llamosas et al. [32], Rodríguez-Martínez et al. [5] and Tremocoldi et al. [4] in avocado by-products extracted via other methods. Trujillo-Mayol et al. compared the antioxidant activity of avocado peel extracts obtained using maceration (12 h at room temperature), ultrasound bath (60 °C, 15 min), microwave (500 W, 120 min) and ultrasound–microwave techniques. DPPH results were around 0.77 mg TE/g d.w., and FRAP values were about 0.16 mg TE/g d.w., with no significant differences recorded between the methods used [33]. These results were much lower than those the reported in this work, which could indicate that sonotrode allowed us to obtain higher antioxidant results from avocado peel and seeds. The better performance of the sonotrode in comparison to the ultrasound bath was previously reported in other works [34,35].

Moreover, regarding the conditions used in the ultrasound bath, 45 min of treatment was required to reach the amount reported in Table 4. Meanwhile, via sonotrode, the time required for the avocado peel and seed to reach higher extracting yields was reduced to 30 min. Moreover, in the sonotrode extraction, the sonde was introduced into direct contact with the extracting solvent and the sample, though during extraction via bath, this action did not happen. In the ultrasound bath, the cavitation was transmitted from water through plastic to the solvent of extraction, attenuating the intensity of the treatment via energy losses [36]. This, this process makes the sonotrode a better election than the ultrasound bath in time, energetic and economic terms. Moreover, sonotrode technology is scalable at the pilot and industrial levels.

As can be seen in Table 4, avocado peel was found to have a total procyanidin content 20% higher than that of avocado seed with the optimal conditions stablished. In contrast, avocado seed was demonstrated to have significantly (*p* < 0.05) higher antioxidant activity based on the three methods measured (Figure S2). It can be explained by the presence of other phenolic compounds in the extracts, as reported by López-Cobo et al. [13], which all have roles in the antioxidant activity. Moreover, as reported by Sálazar-López et al., the polymerization degree of the procyanidins also directly affects the antioxidant activity, making dimers and trimers act as more effective superoxide anion stabilizers than monomers [37]. Thus, as shown in Table 4, avocado peel extract was richer in monomer, while avocado seed extract was richer in dimer and trimer, which could affect the values of the antioxidant activity reported in this paper. Furthermore, it was statistically confirmed (Figure S3) that a lower correlation was found between the monomer and the antioxidant assays (r = 0.0786–0.1193, *p* > 0.05) than between the dimers and trimers (r = 0.5881–0.9735, *p* < 0.05).

## 4. Conclusions

Two Box–Behnken designs were performed to optimize the extraction of flavan-3-ols with high antioxidant activity from avocado peel and seed via sonotrode ultrasound-assisted extraction. The results of this study show that by applying sonotrode extraction, it is possible to increase the flavan-3-ols recovery by about 54% and the antioxidant activity by around 62–76% compared using ultrasound bath technology. In addition, the distributions of flavan-3-ols according to the degree of polymerization between the two avocado by-products were compared. As far as we are concerned, ultrasound-assisted extraction via sonotrode was used for the first time for the obtention of enriched avocado by-products extracts in procyanidins, and it demonstrated itself to be a useful method to take advantage of the flavan-3-ols present in avocado by-products. Furthermore, sonotrode ultrasound technology could be scaled-up to pilot and industrial scales, which would allow us to obtain avocado by-product extracts that could be used as functional ingredients for nutraceutical and food scopes.

## Figures and Tables

**Table 1:** Avocado peel and seed Box–Behnken designs with the natural and coded values (parenthesis) of the extraction conditions and the experimental results obtained for total procyanidins, as well as the antioxidant assays (DPPH, ABTS and FRAP) expressed via the average and the standard deviation.

	Independent Factors			Dependent Factors							
				Avocado Peel				Avocado Seed			
Run	Ethanol (%)	Time (min)	Amplitude (%)	Total Procyanidins (mg CE/g d.w.)	DPPH (mg TE/g d.w.)	ABTS (mg TE/g d.w.)	FRAP (mg TE/g d.w.)	Total Procyanidins (mg CE/g d.w.)	DPPH (mg TE/g d.w.)	ABTS (mg TE/g d.w.)	FRAP (mg TE/g d.w.)
1	100 (1)	5 (−1)	60 (0)	1.05 ± 0.02	0.31 ± 0.00	2.07 ± 0.03	1.15 ± 0.00	4.29 ± 0.05	2.22 ± 0.04	4.08 ± 0.07	2.53 ± 0.12
2	30 (−1)	5 (−1)	60 (0)	6.53 ± 0.01	7.62 ± 0.07	5.18 ± 0.01	10.11 ± 0.12	12.18 ± 0.06	16.88 ± 0.08	16.12 ± 0.05	17.11 ± 0.20
3	100 (1)	45 (1)	60 (0)	2.93 ± 0.05	0.73 ± 0.01	8.44 ± 0.10	1.96 ± 0.02	8.88 ± 0.07	8.26 ± 0.05	6.98 ± 0.14	9.39 ± 0.01
4	30 (−1)	45 (1)	60 (0)	8.03 ± 0.03	7.80 ± 0.05	5.83 ± 0.03	11.09 ± 0.06	13.05 ± 0.09	13.95 ± 0.12	4.06 ± 0.22	11.56 ± 0.09
5	100 (1)	25 (0)	20 (−1)	1.89 ± 0.11	0.37 ± 0.00	1.17 ± 0.04	1.05 ± 0.02	5.13 ± 0.14	1.92 ± 0.02	1.93 ± 0.09	2.36 ± 0.11
6	30 (−1)	25 (0)	20 (−1)	8.83 ± 0.06	9.23 ± 0.08	10.35 ± 0.07	11.00 ± 0.01	13.81 ± 0.18	16.78 ± 0.09	20.68 ± 0.13	19.41 ± 0.02
7	100 (1)	25 (0)	100 (1)	3.71 ± 0.01	0.70 ± 0.02	1.20 ± 0.00	1.67 ± 0.09	8.35 ± 0.12	5.87 ± 0.03	1.29 ± 0.10	6.50 ± 0.10
8	30 (−1)	25 (0)	100 (1)	11.44 ± 0.10	8.68 ± 0.02	5.62 ± 0.11	11.38 ± 0.02	12.52 ± 0.08	13.63 ± 0.03	12.29 ± 0.04	15.86 ± 0.08
9	65 (0)	5 (−1)	20 (−1)	9.19 ± 0.08	2.58 ± 0.09	6.33 ± 0.07	8.30 ± 0.04	9.95 ± 0.04	10.31 ± 0.10	7.51 ± 0.09	11.48 ± 0.01
10	65 (0)	45 (1)	20 (−1)	13.32 ± 0.02	10.80 ± 0.04	23.12 ± 0.06	11.65 ± 0.10	12.61 ± 0.08	15.75 ± 0.07	15.22 ± 0.02	14.27 ± 0.06
11	65 (0)	5 (−1)	100 (1)	13.98 ± 0.11	10.58 ± 0.08	16.09 ± 0.12	14.04 ± 0.03	15.11 ± 0.03	16.89 ± 0.01	25.77 ± 0.08	20.16 ± 0.20
12	65 (0)	45 (1)	100 (1)	16.64 ± 0.09	12.15 ± 0.10	11.03 ± 0.10	11.72 ± 0.04	16.09 ± 0.11	16.88 ± 0.12	21.43 ± 0.15	24.82 ± 0.03
13	65 (0)	25 (0)	60 (0)	20.02 ± 0.01	12.29 ± 0.04	20.07 ± 0.07	14.42 ± 0.10	15.86 ± 0.12	17.10 ± 0.08	24.19 ± 0.01	22.77 ± 0.05
14	65 (0)	25 (0)	60 (0)	19.87 ± 0.03	12.19 ± 0.11	19.14 ± 0.04	14.79 ± 0.02	15.65 ± 0.02	17.11 ± 0.06	22.86 ± 0.07	22.83 ± 0.01
15	65 (0)	25 (0)	60 (0)	20.02 ± 0.00	12.17 ± 0.07	18.80 ± 0.02	14.55 ± 0.08	15.56 ± 0.04	16.70 ± 0.07	23.51 ± 0.10	22.21 ± 0.02

**Table 2:** Estimated regression coefficients of the adjusted second-order polynomial equations and analysis of variance (ANOVA) of avocado peel and seed sonotrode models.

Regression Coefficients	Responses															
	Avocado Peel								Avocado Seed							
	Procyanidins (mg CE/g d.w.)		DPPH (mg TE/g d.w.)		ABTS (mg TE/g d.w.)		FRAP (mg TE/g d.w.)		Procyanidins (mg CE/g d.w.)		DPPH (mg TE/g d.w.)		ABTS (mg TE/g d.w.)		FRAP (mg TE/g d.w.)	
	Effect	*p* Value	Effect	*p* Value	Effect	*p* Value	Effect	*p* Value	Effect	*p* Value	Effect	*p* Value	Effect	*p* Value	Effect	*p* Value
β_0_	8.1298	0.0000 *	5.9622	0.0000 *	8.0353	0.0006 *	7.9278	0.0000 *	10.9987	0.0000 *	11.6117	0.0000 *	11.4461	0.0003 *	12.955	0.0001 *
Lineal																
β_1_	−5.9731	0.0001 *	−7.5989	0.0000 *	−2.4340	0.0385 *	−9.3072	0.0002 *	−6.1622	0.0004 *	−10.7447	0.0002 *	−7.9975	0.0038 *	−9.988	0.0007 *
β_2_	2.2608	0.0008 *	1.8313	0.0007 *	4.2973	0.0128 *	0.7649	0.0319 *	2.4261	0.0023 *	2.1343	0.0059 *	−2.4943	0.0369 *	1.676	0.0224 *
β_3_	2.8280	0.0005 *	1.4863	0.0010 *	−1.9543	0.0579	1.3028	0.0114 *	2.0851	0.0031 *	1.5498	0.0123 *	1.0683	0.1625	3.402	0.0056 *
Crossed																
β_12_	0.1898	0.15512	0.1202	0.1956	2.8571	0.0494 *	−0.0866	0.6902	1.8613	0.0071 *	4.4914	0.0027 *	7.4787	0.0077 *	6.205	0.0030 *
β_13_	−0.3980	0.0427 *	0.4422	0.0196 *	2.3784	0.0691	0.1210	0.5857	2.2557	0.0048 *	3.5437	0.0043 *	3.8723	0.0279 *	3.846	0.0078 *
β_23_	−0.7402	0.0129 *	−3.3287	0.0004 *	−10.9273	0.0036 *	−2.8379	0.0044 *	−0.8383	0.0334 *	−2.7257	0.0072 *	−6.0253	0.0118 *	0.933	0.1123
Quadratic																
β_11_	11.0757	0.0000 *	6.1931	0.0000 *	11.7574	0.0009 *	6.8304	0.0002 *	4.7850	0.0003 *	6.0232	0.0004 *	12.0744	0.0008 *	9.550	0.0003 *
β_22_	4.2587	0.0001 *	1.9114	0.0003 *	2.2010	0.0235 *	1.6789	0.0034 *	1.3009	0.0039 *	0.6176	0.0365 *	3.6357	0.0088 *	2.903	0.0037 *
β_33_	2.4284	0.0003 *	1.2775	0.0007 *	2.9960	0.0129 *	1.4813	0.0043 *	0.9486	0.0074 *	1.3943	0.0075 *	2.3983	0.0200 *	2.016	0.0077 *
R^2^	0.9996		0.9997		0.9635		0.9971		0.9907		0.9885		0.9878		0.9933	
*p* model	0.0000 *		0.0014 *		0.0031 *		0.0000 *		0.0004 *		0.0018 *		0.0212 *		0.0058 *	
*p* lack of fit	0.1551		0.1956		0.1279		0.2794		0.0899		0.0762		0.1625		0.1123	

* Significant at α ≤ 0.05. Subscripts after β: 1, ethanol (%); 2, time (min); 3, amplitude (%).

**Table 3:** Optimal conditions selected and the predicted and obtained values expressed as the mean and standard deviation of the avocado peel and seed sonotrode models.

**Parameters**	**Optimal Conditions**							
	**Avocado Peel**				**Avocado Seed**			
Ethanol (%)	60				55			
Time (min)	30				30			
Amplitude (%)	70				90			
	**Procyanidins**	**DPPH**	**ABTS**	**FRAP**	**Procyanidins**	**DPPH**	**ABTS**	**FRAP**
Predicted Value (mg/g d.w.)	20.73 ± 0.59	13.45 ± 0.44	19.20 ± 2.23.5	15.14 ± 0.63	16.99 ± 0.57	17.99 ± 0.83	25.41 ± 2.39	25.47 ± 1.24
Obtained value (mg/g d.w.)	20.80 ± 0.10	13.80 ± 0.22	20.23 ± 0.12	14.97 ± 0.25	16.70 ± 0.20	17.76 ± 0.15	25.87 ± 0.33	26.62 ± 0.92
CV (%)	0.23	1.81	3.69	0.78	1.22	0.91	1.26	3.12
Control (mg/g d.w.)	9.66 ± 0.24	5.26 ± 0.11	6.99 ± 0.34	5.00 ± 0.22	7.64 ± 0.34	5.36 ± 0.26	6.12 ± 0.19	8.00 ± 0.19

**Table 4:** Distribution according to the polymerization degree of flavan-3-ols in avocado peel and seed optimal extracts obtained via sonotrode and ultrasound bath controls expressed as the mean and standard deviation.

	Avocado Peel (mg/g d.w.)		Avocado Seed (mg/g d.w.)	
	Sonotrode	Ultrasound Bath	Sonotrode	Ultrasound Bath
Monomer	8.17 ± 0.04	4.53 ± 0.06	0.72 ± 0.05	0.48 ± 0.05
dp2	1.40 ± 0.01	0.76 ± 0.02	4.58 ± 0.02	2.68 ± 0.08
dp3	1.15 ± 0.00	0.91 ± 0.04	1.65 ± 0.03	1.09 ± 0.06
dp4	1.09 ± 0.02	0.91 ± 0.03	1.09 ± 0.02	0.68 ± 0.04
dp5	0.90 ± 0.01	0.68 ± 0.01	0.75 ± 0.01	0.46 ± 0.03
dp6	0.54 ± 0.00	0.49 ± 0.01	0.64 ± 0.01	0.38 ± 0.01
dp7	0.48 ± 0.00	0.29 ± 0.01	0.48 ± 0.01	0.29 ± 0.02
dp8	0.24 ± 0.00	0.16 ± 0.00	0.35 ± 0.00	0.21 ± 0.00
dp9	0.06 ± 0.00	0.07 ± 0.00	0.23 ± 0.00	0.15 ± 0.00
dp10	0.03 ± 0.00	0.02 ± 0.00	0.15 ± 0.00	0.09 ± 0.00
Polymer	6.72 ± 0.03	0.83 ± 0.06	6.03 ± 0.05	1.15 ± 0.10
Total procyanidins	20.80 ± 0.10	9.66 ± 0.24	16.69 ± 0.20	7.64 ± 0.34

## Data Availability

Data sharing not applicable.

## Supplementary Materials

The following supporting information can be downloaded at: https://www.mdpi.com/article/10.3390/antiox12071409/s1, Figure S1. Fluorescence chromatograms of the flavan-3-ols identified in avocado peel and seed extracts obtained via sonotrode optimal conditions. Cat + epicat: catechin and epicatechin; dp: degree of polymerization. Figure S2. Representation of the antioxidant activity (DPPH, ABTS and FRAP) of the optimal extracts obtained via sonotrode in comparison to the ultrasound bath. Different letters (a–d) in the same method indicate significative differences. Figure S3. Correlation heatmap of the procyanidin profile and antioxidant assays.
