# Supplementary material for: Response Surface Methodology for the Optimization of Flavan-3-ols Extraction from Avocado By-Products via Sonotrode Ultrasound-Assisted Extraction
Source: Antioxidants (Basel). 2023 Jul 11;12(7):1409. doi: 10.3390/antiox12071409 (PMC10376872; doi:10.3390/antiox12071409)
Supplement: Supplementary file 1 [file antioxidants-12-01409-s001.zip › antioxidants-2429631-supplementary.pdf]

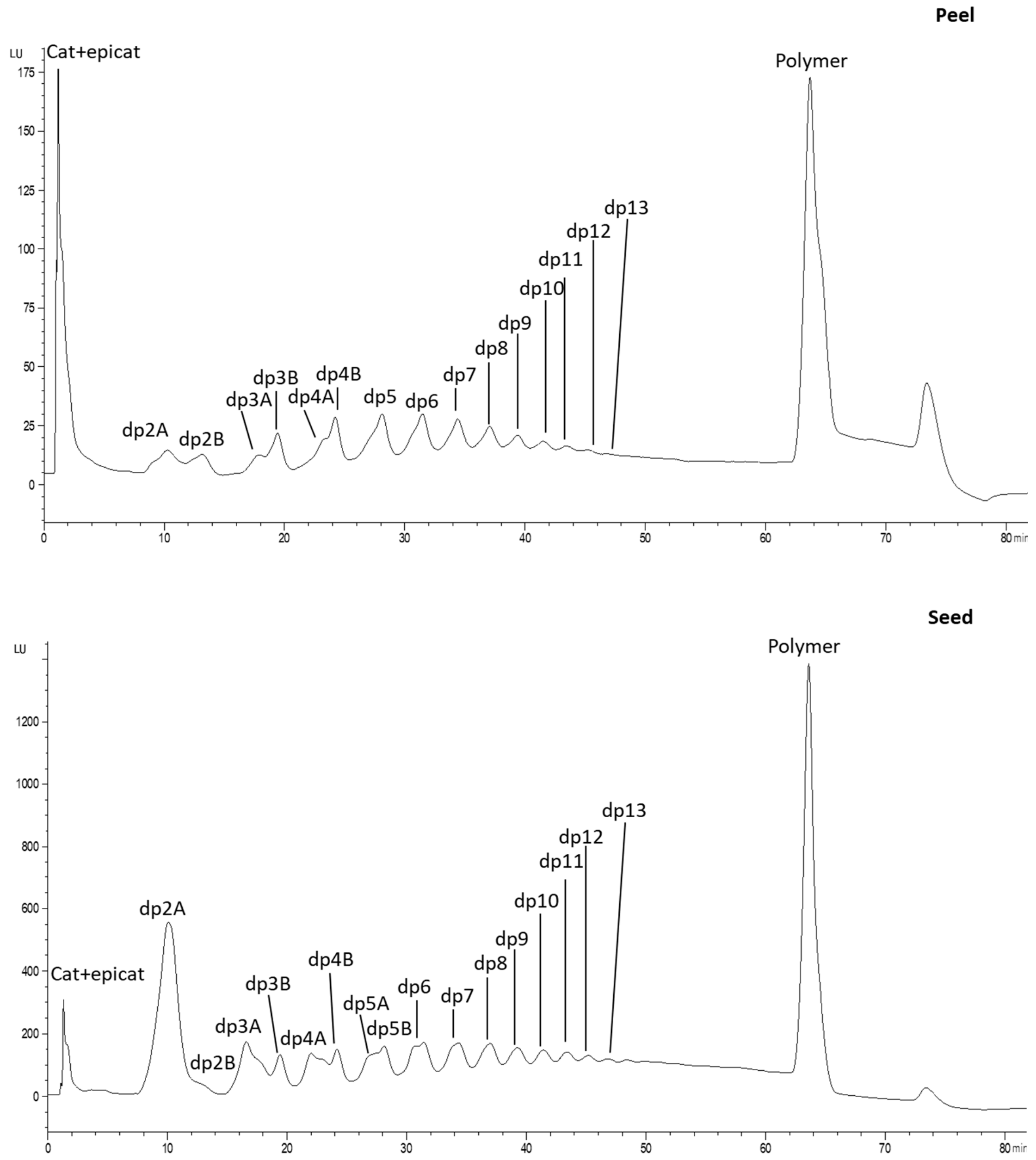

Figure S1. Fluorescence chromatograms of the identified flavan-3-ols in avocado peel and seed extracts obtained by sonotrode optimal conditions. Cat+epicat: catechin + epicatechin; dp: degree of polymerization.

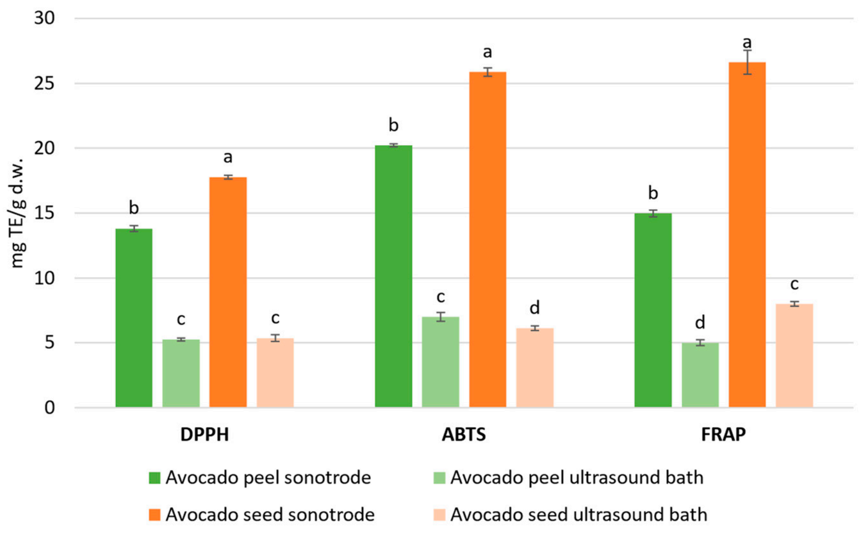

Figure S2. Representation of the antioxidant activity (DPPH, ABTS and FRAP) of the optimal extracts obtained by sonotrode in comparison with ultrasound bath. Different letters (a-d) in the same method indicate significative differences.

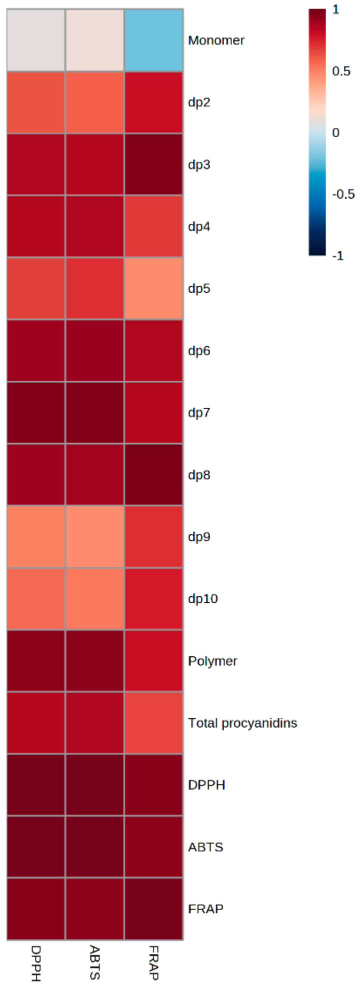

Figure S3. Correlation heatmap of the procyanidin profile and the antioxidant assays in the avocado by-products.
